# Changes in the Progression of Chronic Kidney Disease in Patients Undergoing Fecal Microbiota Transplantation

**DOI:** 10.3390/nu16081109

**Published:** 2024-04-10

**Authors:** Giovanna Yazmín Arteaga-Muller, Samantha Flores-Treviño, Paola Bocanegra-Ibarias, Diana Robles-Espino, Elvira Garza-González, Graciela Catalina Fabela-Valdez, Adrián Camacho-Ortiz

**Affiliations:** 1 Department of Nephrology, University Hospital Dr. José Eleuterio González, Autonomous University of Nuevo Leon, Monterrey 64460, Nuevo Leon, Mexico; giomuller@gmail.com; 2 Department of Infectious Diseases, University Hospital Dr. José Eleuterio González, Autonomous University of Nuevo Leon, Monterrey 64460, Nuevo Leon, Mexico; samflorest@gmail.com (S.F.-T.); paola.bocanegraib@gmail.com (P.B.-I.); grace.fabela@hotmail.com (G.C.F.-V.); 3 Department of Clinical Pathology, University Hospital Dr. José Eleuterio González, Autonomous University of Nuevo Leon, Monterrey 64460, Nuevo Leon, Mexico; drobesp@hotmail.com; 4 Department of Biochemistry, School of Medicine, Autonomous University of Nuevo Leon, Monterrey 64460, Nuevo Leon, Mexico; elvira_garza_gzz@yahoo.com; 5 Department of Infectious Diseases and Hospital Epidemiology, University Hospital Dr. José Eleuterio González, Autonomous University of Nuevo Leon, Monterrey 64460, Nuevo Leon, Mexico

**Keywords:** chronic kidney disease, fecal microbiota transplant, disease progression

## Abstract

Chronic kidney disease (CKD) is a progressive loss of renal function in which gut dysbiosis is involved. Fecal microbiota transplantation (FMT) may be a promising alternative for restoring gut microbiota and treating CKD. This study evaluated the changes in CKD progression in patients treated with FMT. Patients with diabetes and/or hypertension with CKD clinical stages 2, 3, and 4 in this single-center, double-blind, randomized, placebo-controlled clinical trial (NCT04361097) were randomly assigned to receive either FMT or placebo capsules for 6 months. Laboratory and stool metagenomic analyses were performed. A total of 28 patients were included (15 FMT and 13 placebo). Regardless of CKD stages, patients responded similarly to FMT treatment. More patients (53.8%) from the placebo group progressed to CKD than the FMT group (13.3%). The FMT group maintained stable renal function parameters (serum creatinine and urea nitrogen) compared to the placebo group. Adverse events after FMT treatment were mild or moderate gastrointestinal symptoms. The abundance of Firmicutes and Actinobacteria decreased whereas Bacteroidetes, Proteobacteria and *Roseburia* spp. increased in the FMT group. CKD patients showed less disease progression after FMT administration. The administration of oral FMT in patients with CKD is a safe strategy, does not represent a risk, and has potential benefits.

## 1. Introduction

Chronic kidney disease (CKD) is a progressive loss of renal function that leads to an accumulation of uremic toxins [1] and a potential increased risk of developing cardiovascular disease, mainly due to type 2 diabetes, hypertension, obesity, and aging. Worldwide, up to one million deaths are estimated to be associated with CKD [2]. Although CKD has high morbidity and mortality, treatment options are limited [3]. Current CKD treatment guidelines focus on preventing disease progression. Pharmacologic interventions include renin-angiotensin-aldosterone system blockers, statins, and control of the underlying disease and blood pressure [4].

The gut microbiota has protective, structural, and metabolic functions that help maintain a healthy intestinal homeostasis [5]. An imbalance in the microbiological community, also called dysbiosis, is involved in the progression of chronic diseases, including CKD, diabetes mellitus, and arterial hypertension, among others [6]. Alterations in the gut microbiota (i.e., dysbiosis), particularly a decrease in bacterial diversity, can be associated with CKD. An increase in pro-inflammatory and uremic metabolite-producing bacteria and a decrease in anti-inflammatory-producing bacteria contribute to disease progression [7]. In patients with CKD, uremia alters the composition and metabolism of the gut microbiota [8]. Gut microbiota composition can also contribute to the development of other diseases such as immunological and metabolic disorders [9]. Targeting gut microbiota at all stages of CKD can be used to restore gut microbiota to improve kidney function [7]. Thus, restoring the healthy gut microbial community is an optimistic therapeutic strategy in diseases associated with gut dysbiosis [10,11].

Fecal microbiota transplantation (FMT) is the infusion of a fecal suspension recovered from a healthy donor directly into the gut of a recipient [11]. The administration of the fecal suspension can be nasogastrically, colonoscopically, or orally. FMT is used primarily for the treatment of recurrent *Clostridioides difficile* infections with a high success rate and safety [11]. FMT is also proposed as an emerging treatment for several gastrointestinal and non-gastrointestinal disorders, including inflammatory bowel disease, metabolic syndrome, Parkinson’s disease, multiple sclerosis, obesity, insulin resistance, and autism, among others [11,12]. FMT is currently the only microbe-based therapy which allows us to transfer a complex gut ecosystem [7]. Nevertheless, few clinical studies using FMT to treat CKD are currently available, and preliminary data suggest that FMT may be a promising alternative for treating CKD [3]. This study evaluated the changes in CKD progression and the safety of the intervention in patients treated with FMT in addition to their standard of care in a tertiary-care hospital in Mexico.

## 2. Materials and Methods

### 2.1. Study Design

This study was a single-center double-blind, randomized, placebo-controlled clinical trial (www.clinicaltrials.gov; accessed on 18 January 2024; NCT04361097). Participants were randomly assigned in a 1:1 ratio to receive either FMT or placebo for 6 months. Neither the patients nor the clinicians performing patient evaluations were aware of group assignments. Participants received dietary instructions during the scheduled interviews. Information about the quality of life was obtained by the Dartmouth Coop Functional Health Assessment/World Organization of National Colleges, Academies and Academic Association of General Practitioners (COOP/WONCA) functional health assessment test [13]. Screening for possible adverse effects was performed on days 0, 10, 30, 90, and 180.

### 2.2. Study Site

The study was performed at the Dr. José Eleuterio González University Hospital, an academic hospital with an average of 1500 CKD outpatient consultations per year. The setting was a hemodialysis unit with 20 hemodialysis machines with an average of 12,500 hemodialysis sessions per year.

### 2.3. Study Groups

**Figure 1:**
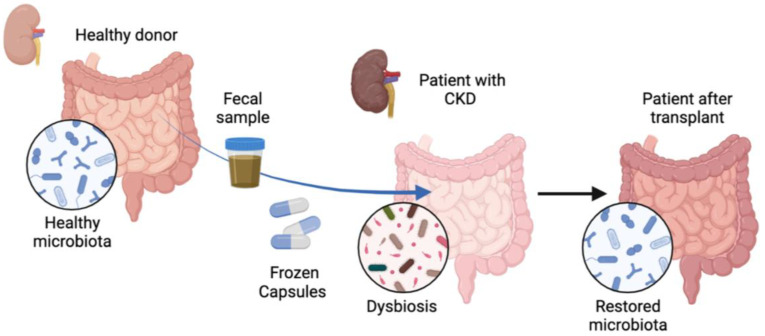
Treatment of patients with chronic kidney disease with fecal microbiota transplantation. Patients (n = 28) with chronic kidney disease (CKD) were treated orally with frozen capsules of fecal microbiota transplantation (FMT) obtained from a healthy donor or with placebo capsules for 6 months. Gut dysbiosis was observed in patients prior to FMT administration, which changed after FMT treatment and healthy microbiota was restored.

Patients with CKD in clinical stages according to the Kidney Disease Improving Global Outcomes (KDIGO) classification, aged between 18 and 80 years, were invited to participate. Excluded patients included those with a malignant tumor in which their last treatment was less than 5 years previously, those who had received antibiotics for any reason during the month before enrollment, those who had received probiotics in the past 3 months, those who were diagnosed with *Clostridioides difficile* infection in the past year, those who had previously undergone FMT, those who had presented exacerbations of CKD during the 3 months before enrollment; and if the clinician anticipated that the patient would undergo renal replacement therapy in the following 6 months. After the patients signed the written informed consent, they were randomized in a blind manner in a 1:1 ratio to receive either FMT or placebo capsules (Figure 1).

### 2.4. Selection of FMT Donors

A bank of feces from donors previously evaluated is currently stored in the Laboratory of Infectious Diseases of the University Hospital [14]. Donors were of either sex and over 18 years of age, non-pregnant, with a body mass index of 20–25 kg/m^2^, with normal total blood count and normal liver enzymes serum levels. Exclusion criteria were consumption of proton-pump inhibitors, antibiotics, immunosuppressive medication, hospitalization, and diarrhea 3 months prior to donation. Additional exclusion criteria were high-risk sexual behavior, having a first-degree relative with diabetes mellitus, abdominal surgery, and any gastrointestinal disease or cancer.

### 2.5. Screening of FMT Donors

Blood samples were taken from the potential donors prior to fecal sample collection. A complete blood count (CBC) test was performed to exclude those participants with abnormal results. Serological tests for hepatitis A, hepatitis B, hepatitis C, human immunodeficiency virus (HIV) type 1 and 2, *Trypanosoma cruzi*, *Brucella* spp., and *Treponema pallidum* were performed on all potential donors. The presence of cytomegalovirus and Epstein–Barr virus was also assessed in blood samples from potential donors by real-time PCR (TIB BIOMOL LightMix Kit, Roche, Mannheim, Germany). Stool specimens were collected, and ova and parasite examination, bacterial culture, and a stool antigen test for *Helicobacter pylori* were performed. Real-time PCR analysis was performed using BioFire FilmArray Gastrointestinal Panel (BioFire Diagnostics, Salt Lake City, UT, USA) to detect enteropathogenic pathogens such as *Campylobacter* spp. (*C. jejuni*, *C. coli* and *C. upsaliensis*), *C. difficile*, *Plesiomonas shigelloides*, *Salmonella* spp., *Yersinia enterocolitica*, *Vibrio* spp. (*V. parahaemolyticus*, *V. vulnificus* and *V. cholerae*), *Escherichia coli* (enteroaggregative, enteropathogenic, enterotoxigenic, Shiga-like toxin-producing, O157, *Shigella*/enteroinvasive), *Cryptosporidium* spp., *Cyclospora cayetanensis*, *Entamoeba histolytica*, *Giardia lamblia*, adenovirus, astrovirus, norovirus, rotavirus, and sapovirus. Fecal samples were also screened for the presence of drug-resistant bacteria, such as carbapenemase-resistant Enterobacterales (CRE). After DNA was extracted from stool specimens using a DNeasy PowerSoil kit (QIAGEN, Hilden, Germany), carbapenemase-encoding genes (KPC, VIM, IMP, NDM, OXA-48, TEM, SHV, CTX-M, CYM, and *mcr*-1) were amplified by end-point PCR [15]. The detection of severe acute respiratory syndrome coronavirus 2 (SARS-CoV-2) RNA in stool samples by real-time PCR was not necessary as the samples were collected before the year 2019.

### 2.6. Preparation of FMT Capsules

From each donor, three stool samples were collected within 2 weeks after the initial donor evaluation. Fresh stool was resuspended in 0.9% saline solution and filtered three times through a sterile gauze to remove particles > 330 μm. Then, 15% (*v*/*v*) glycerol was added as a bacterial cryoprotectant. Subsequently, 500 µL of fecal solution (FMT capsules) or saline solution (placebo capsules) was deposited into commercially available gelatin capsules (Encapsuladoras Mexico, S.A. de C.V, Chihuahua, Mexico), first into size 0 capsules and then into size 00 capsules. Capsules were stored frozen at −80 °C. Each FMT capsule contained 0.5 g/mL of fecal matter, which corresponded to 4.3 × 10^10^ of total bacterial cells per g of fecal matter).

### 2.7. Administration of FMT or Placebo Capsules

Participants in both groups (FMT or placebo) received 15 orally administered capsules every 12 h four times every dosing period. Dosing periods were administered on days 0, 10, and 30. Every patient received 180 capsules of either FMT or placebo during the study. Each 15-capsule dosage was orally administered in less than 1 h. Scheduled patient visits were arranged on days 0, 10, 30, 90, 120, and 180 after randomization. Laboratory analyses were measured during the patients’ scheduled visits. They consisted of 24-h urine protein and creatinine clearance, serum creatinine, blood urea nitrogen, bicarbonate, phosphorus, cell blood count, and C-reactive protein (CRP). Stool samples were collected for genomic analysis on days 0, 30, and 90.

### 2.8. Metagenomic Analysis of Gut Microbiome

Eight patients from the FMT treatment group and six from the placebo group were selected for metagenomic analysis. Feces collected before the treatment (day 0) and on days 30 and 90 after FMT or placebo treatment were analyzed. DNA from stool specimens was extracted using the DNeasy PowerSoil kit (Qiagen, Hilden, Germany). The samples were processed and analyzed using a MiSeq instrument (Illumina Inc, San Diego, CA, USA) at Molecular Research LP (Shallowater, TX, USA), following the manufacturer’s protocol. The V4 region of the 16S rRNA gene of each sample was amplified using previously described primers [16]. All amplicon products were purified using calibrated SPRI beads. Samples were sequenced utilizing the Illumina NovaSeq chemistry following the manufacturer’s protocol. The resulting Q25 sequence data were processed using a proprietary analysis pipeline (http://www.mrdnalab.com, accessed on 6 August 2021, MR DNA, Shallowater, TX, USA). Sequences were denoised and depleted of barcodes, primers, and chimeras. Operational taxonomic units (OTUs) were defined as clustering at 3% divergence (97% similarity), classified using BLASTn against a curated NCBI database, and compiled into each taxonomic level. Alpha diversity was defined as the diversity within a specific area. Beta diversity was defined as the analysis of the microbial community structure.

### 2.9. Statistical Analysis

Using hypothesis testing and the difference of two proportions for independent groups with an α value of 0.05, a β error of 0.2, a power of 80%, and a standard deviation of 0.5, 15 subjects per group were needed. Continuous variables were described as mean and standard deviations; percentages and frequencies were used for categorical variables. A Mann–Whitney U test or Student’s *t*-test was used to compare means. A Chi-square or Fisher’s exact test was used to compare proportions. IBM SPSS version 20 (IBM Corp., Armonk, NY, USA) was used. In metagenomic analyses, statistical analysis was performed using XLstat version 2021.1, NCSS 2007, “R”, and NCSS 2010. Alpha and beta diversity analysis was conducted as previously described using Qiime 2 [16]. Statistical comparisons were conducted using repeated measures ANOVA. Post hoc pairwise comparisons were made using a Tukey’s test. Comparison of the Alpha diversity of samples was assessed using comparisons of observed features (Amplicon Sequence Variants, ASVs) and Shannon Diversity indices, which were conducted using Kruskal–Wallis pairwise comparisons. Beta diversity of samples was analyzed using a weighted UniFrac distance matrix to analyze the microbial community structure. Pairwise analysis of similarities (ANOSIM) was utilized to determine if there were any significant differences between the microbial communities. A *p*-value < 0.05 was considered significant.

## 3. Results

### 3.1. Characteristics of CKD Patients

We screened 273 patients, of whom 84 met the inclusion criteria, and 30 agreed to participate. Two patients were eliminated from the placebo group because they were retrospectively classified as acute-on-chronic renal failure. A total of 28 patients completed the study (15 from the FMT treatment group and 13 from the placebo group). The median age of the FMT group was 57 years vs. 56 for the placebo group. Male gender predominated in the FMT group (61.5% vs. 40% in the placebo group). Type 2 diabetes was the most frequent underlying illness (n = 25, 89.2%), which was more frequent in the FMT group (93.3% vs. 84.6%). Hypertension was observed in three (10.7%) patients (6.6% vs. 15.3%). Patients were classified into CKD stages according to their GFR (glomerular filtration rate) values, which were stage 1 (n = 1), 2 (n = 3), 3a (n = 5), 3b (n = 5), 4 (n = 12), and 5 (n = 2). There were no significant differences in the CKD stages between the groups. Regarding albuminuria stages, 12 patients were on the A2 level and 16 were on A3, and no significant differences were observed between the treatment and placebo groups (Table 1).

All patients included in the study were treated with angiotensin-converting enzyme inhibitors (ACEI) or angiotensin II receptor blockers (ARB). None of the patients from either group received sodium-glucose cotransporter-2 (SGLT2) inhibitors or finerenone. Arterial blood pressure showed no significant differences between the groups or during the study period. No changes were performed to the basal medications of the patients during the study.

### 3.2. Laboratory Analyses from CKD Patients after FMT Treatment

**Figure 2:**
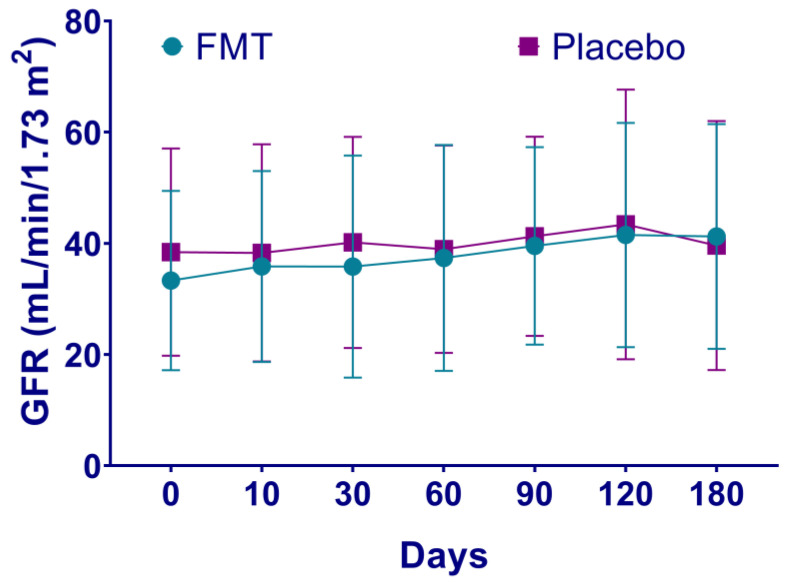
Estimates of glomerular filtration rate of patients with chronic kidney disease after fecal microbiota transplantation. The progression of CKD in patients after either fecal microbiota transplantation (FMT) or placebo treatment is shown, expressed as a decrease in the glomerular filtration rate (GFR) > 1 mL/min/1.73 m^2^. In patients with CKD treatment, the estimated GFR loss is 2.3 to 4.5 mL/min/1.73 m^2^ per year. Rapid progression is defined as a sustained decline in GFR > 5 mL/min/1.73 m^2^ per year [4].

Mean baseline leukocytes levels were higher in patients in the FMT group compared to the placebo group (8.72 vs. 6.81 K/uL, *p =* 0.01, Table 1). Increased leukocytes values continued in the FMT group compared to the placebo group after days 10, 30 and up to 120 (*p <* 0.05, Supplementary Table S1). Glucose levels also increased in the FMT group after day 90 (123.39 vs. 99.54 mg/dL, *p =* 0.04, Supplementary Table S1) and up to 180 days post-treatment (127.23 vs. 94.85 mg/dL, *p =* 0.02, Supplementary Table S1). Blood urea nitrogen showed differences only on day 10 after treatment and was higher than the placebo group (40.93 vs. 30.31 mg/dL, *p* = 0.04, Supplementary Table S1). Uric acid baseline levels were also higher in the FMT group (7.79 vs. 6.27 mg/dL, *p* = 0.02), which decreased from day 10 to day 180 (*p* = 0.02) compared to the placebo group (6.21 vs. 7.28 mg/dL, *p* = 0.04, Supplementary Table S1). CRP was higher in the FMT group compared to the placebo group on day 120 (0.88 vs. 0.57 mg/dL, *p* = 0.04, Supplementary Table S1). In contrast, mean baseline bicarbonate (HCO_3_) values were lower in the FMT group compared to the placebo group (22.98 vs. 26.32 mEq/L, *p* = 0.008), which continued to day 60 (20.99 vs. 24.46 mEq/L, *p* = 0.002, Supplementary Table S1) and to day 180 (20.85 vs. 24.54 mEq/L, *p* = 0.01, Supplementary Table S1). Hemoglobin, platelets, 24-h urine protein, creatinine clearance, serum creatinine, potassium, and phosphorus levels did not show statistical differences between the groups throughout the study. From baseline to day 180, 24-h urine protein increased slightly in the FMT group (2.05 vs. 3.01 g/24 h) compared to the placebo group (1.67 vs. 2.34 g/24 h) and phosphorus levels increased in the placebo group (4.14 vs. 4.39 mg/dL) unlike in the FMT group (4.20 vs. 4.22 mg/dL), although not statistically. The COOP/WONCA test for quality of life did not show differences between the groups. The GFR was lower in the placebo group compared to the FMT group, although not statistically. The estimates of the GFR of patients in the FMT group showed a tendency to increase from the baseline to day 180 (average 34.58 vs. 41.23 mL/min/1.73 m^2^), suggesting lower CKD progression. Instead, the estimates of the GFR of patients in the placebo group did not show a tendency to increase (average 38.43 vs. 39.61 mL/min/1.73 m^2^), suggesting CKD progression. In particular, the progression of CKD expressed as a decrease in the GFR > 1 mL/min/1.73 m^2^ was noted in two (13.3%) patients treated with FMT compared to seven (53.8%) patients in the placebo group (*p =* 0.04, Figure 2).

### 3.3. Adverse Events after FMT Treatment

The adverse events (AEs) that occurred in the FMT group compared to the placebo group were abdominal distention, diarrhea, constipation, increased frequency of bowel movements, and flatulence (Table 1). All AEs were classified as grade 1 (mild) or grade 2 (moderate), and there were no severe adverse events in either group.

### 3.4. Microbiome Analysis

**Figure 3:**
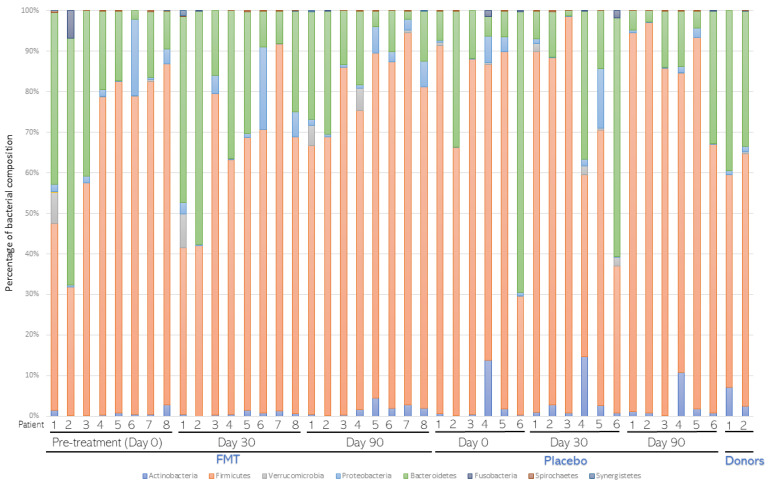
Average bacterial composition at the Phylum level. Stool samples from patients (n = 8) subjected to fecal microbiota transplantation (FMT) or placebo (n = 6) treatments were collected before (Day 0) and 30 (Day 30) and 90 (Day 90) days after treatment. After metagenomic analysis, distribution of bacterial composition at the phylum level was assessed and compared in both groups. The distribution of FMT donors was also compared.

**Figure 4:**
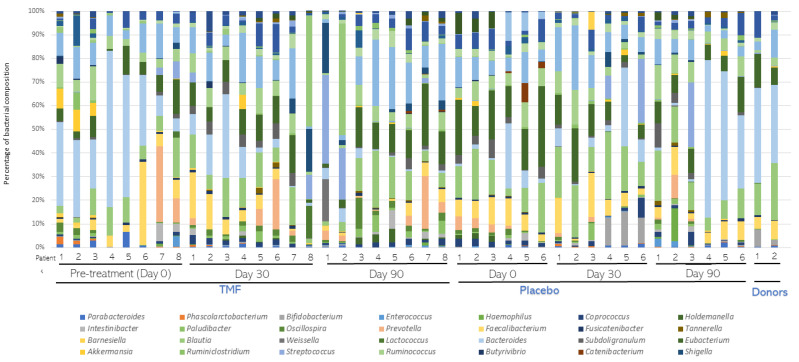
Average bacterial composition at the Genera level. Stool samples from patients (n = 8) subjected to fecal microbiota transplantation (FMT) or placebo (n = 6) treatments were collected before (Day 0) and 30 (Day 30) and 90 (Day 90) days after treatment. After metagenomic analysis, distribution of bacterial composition at the Genera level was assessed and compared in both groups. The distribution of FMT donors was also compared.

A total of 14 patients (8 of the FMT treatment group and 6 of the placebo group) were selected for metagenomic analysis. Average bacterial composition at the phylum level was assessed and compared in both groups (Figure 3). Firmicutes, Bacteroidetes, Actinobacteria, and Proteobacteria were the most abundant phyla in both groups. Few changes were detected among the groups. Overall, the average proportion of Firmicutes and Actinobacteria decreased in the FMT group compared to the placebo group. Instead, the average proportion of Bacteroidetes and Proteobacteria was slightly higher in the FMT group than in the placebo group. Average bacterial composition at the Genera level was also assessed and compared in both groups (Figure 4). Only one genus, *Roseburia* spp., was lower in the FMT group compared to the placebo group after 30 and 90 days of treatment (*p* < 0.0001, Table 2). According to alpha and beta diversity analysis, the microbial diversity and community structure did not differ between the groups (*p* > 0.05, Supplementary Figure S1).

## 4. Discussion

CKD is anticipated to become one of the top global health issues within this century. Diabetes, hypertension, or other causes of CKD, result in progressive and irreversible nephron loss, reduced renal regenerative capacity, metabolic changes, and inflammation, ultimately leading to fibrosis [17]. The role of the gut microbiota in the development and progression of CKD involves provoking inflammation, proteinuria, hypertension, and kidney disease [9,18,19]. Short-chain fatty acids (SCFAs) such as butyrate, acetate, and propionate are gut microbiota-derived metabolites produced by the saccharolytic fermentation of non-digestible compounds. These products are produced mainly by Firmicutes and Bacteroidetes [20,21]. The entry of retained waste products (e.g., urea) into the intestinal lumen can modify microbiota composition [21]. In CKD, the increase in urea levels induces an increase in bacteria with proteolytic activity (responsible for producing uremic toxins) and a decrease in bacteria with saccharolytic activity [6,17]. Uremic toxins, such as indoxyl sulfate (IS), p-cresol sulfate (pCS), and trimethylamine-N-oxide (TMAO), can promote chronic inflammation and contribute to kidney disease progression [1,21]. In patients with end-stage renal disease, the abundance of bacterial families expressing butyrate-producing enzymes decreased whereas the abundance of bacterial families expressing urease and uricase, as well as IS and pCS-producing enzymes, increased [22]. The gut–kidney axis can be affected by gut microbiota. The renin–angiotensin system (RAS) is strongly associated with gut microbiota and CKD. Uremic toxins can activate the RAS, provoking gut dysbiosis and contributing to CKD progression. A therapeutic approach for CKD treatment might be the suppression of RAS activation, modulated by gut microbiota [3]. Therefore, changes in the gut microbiota could improve the renal function by decreasing uremic toxins and fibrosis, also based on studies where probiotics, prebiotics, and fibers were administered [17].

The restoration of the healthy gut microbial community in diseases associated with dysbiosis might be a therapeutic strategy for the prevention and treatment of CKD [9,10,11]. Indeed, FMT therapy may be a promising alternative for restoring beneficial gut microbiota and treating CKD [3,7]. Previous studies in mice models with diabetic kidney disease or CKD show that FMT administration prevented body weight gain, reduced albuminuria and intestinal inflammation, improved insulin resistance [23], and delayed CKD development by altering gut microbiota [24]. Two previous clinical case studies showed improved kidney function (increase in serum protein and albumin levels and decrease in 24-h urine protein) after FMT treatment [25,26]. These data suggest that FMT treatment can alter the gut microbiota composition and might be a promising therapeutic choice for CKD [3]. Nevertheless, more studies are needed to evaluate the efficacy of FMT on diseases associated with dysbiosis [27].

Regarding prevention and treatment of IgA nephropathy, which is an immune-complex-mediated glomerular disease, targeting gut microbiota might be a promising therapeutic approach. A previous study used bi-directional Mendelian randomization to explore the causal relationship between gut microbiota and IgA nephropathy. *Enterorhabdus*, *Peptococcaceae*, and *Prevotellaceae* correlated with genetic human-leukocyte-antigen (HLA) and reduced the risk of IgA nephropathy. Instead, *Butyricicoccus* represented a risk factor for this disease. Therefore, these bacterial taxa could be used to predict the development of IgA nephropathy [28].

In our study, we compared the changes in CKD progression and the safety of the intervention in 28 patients with either diabetes or hypertension treated either with FMT or a placebo. Our results show that regardless of CKD stages, patients responded similarly to FMT treatment. More patients (53.8%) from the placebo group progressed to CKD compared to the FMT group (13.3%). The FMT group maintained stable renal function parameters, such as serum creatinine and urea nitrogen, compared to the placebo group. Based on 24-h urine creatinine clearance levels after FMT administration, an improvement in renal function was observed as opposed to the expected progressive decline in CKD with proteinuria. The KDIGO group defines CKD progression as a decrease in the CKD stage, or a reduction of 25% or more from the baseline function, as a result of the evaluation of GFR and albuminuria [4]. KDIGO also states that small fluctuations in GFR are relatively common and do not necessarily indicate disease progression. However, these fluctuations could indicate chronic inflammation in CKD patients, where the intestinal microbiome is severely altered. Thus, these guidelines may need revision to consider these issues.

Gut dysbiosis in CKD is characterized by an increase in Proteobacteria and Fusobacteria phyla and the *Enterobacteriaceae* family and a decrease in *Roseburia*, *Faecalibacterium*, and *Prevotella* genera [29]. In our study, all CKD patients presented intestinal dysbiosis before FMT treatment, influencing the accumulation of uremic toxins. After FMT treatment, the abundance of Firmicutes and Actinobacteria phyla decreased in the FMT group compared to the placebo group, whereas the abundance of Bacteroidetes and Proteobacteria increased. Abundance of *Roseburia* spp. was observed to decrease in CKD patients after 30 and 90 days of FMT treatment. *Roseburia* are obligate Gram-positive anaerobic bacteria that are part of the gut commensal microbiota. This genus was previously reported as a possible health marker due to the production of SCFAs, mainly butyrate, which may contribute to CKD-associated inflammation and disease progression [30,31]. Higher glucose levels, which we observed on day 90 in the FMT group, are related to a lower abundance of *Roseburia* spp. [30]. Our results also show that CRP was higher in the FMT group on day 120, similar to a previous report, in which a high abundance of *Roseburia* spp. correlated with lower CRP concentrations [31].

We acknowledge some limitations of this study, such as the small sample size, in addition to not being able to perform the metagenomic analysis on all the participants. Furthermore, uremic toxins such as IS, pCS, and TMAO, previously described as inflammatory biomarkers, were not measured in the study. In addition, future studies should include a follow-up of CKD patients after FMT treatment up to 12 months of study, in order to provide in-depth analysis of the benefits of FMT over CKD.

This clinical trial also provides long-term safety data for the clinical application of FMT on CKD. Overall, FMT is generally considered safe and is well tolerated. Serious adverse events related to FMT occur in <1% of patients [32]. In our study, adverse events after administering FMT were only mild or moderate (abdominal distention, diarrhea, constipation, increased frequency of bowel movements, and flatulence), and our trial was considered a safe intervention. Serious adverse events associated with FMT can occur with the colonization of drug-resistant microorganisms in donors [33]. Thus, a rigorous strict donor screening is first required to be able to perform FMT on susceptible patients. Regarding our own bank of stools from donors, we assessed FMT samples thoroughly by different methods before FMT administration and following FDA recommendations to reduce the risk of transmitting infectious microorganisms from donors to CKD patients. We discarded the presence of enteropathogenic pathogens and colonization by drug-resistant bacteria, such as carbapenemase-resistant Enterobacterales (CRE).

A single-dose administration of FMT might not be enough to cure CKD complications. It is possible that prolonged FMT consumption might be needed to halt CKD progression. FMT administration might also need to be combined with diet control and pharmacological treatment for CKD progression [7]. As current therapeutic strategies to prevent CKD progression are limited, more therapeutic options should be sought.

## 5. Conclusions

This study represents the first clinical trial of the administration of FMT in frozen capsules to patients with CKD secondary to diabetes and hypertension. CKD patients showed less disease progression at 6 months after FMT administration. Renal function parameters remained stable during the follow-up of patients. Furthermore, FMT treatment with frozen capsules is a safe strategy and does not represent a risk in CKD patients. The possibility that inflammation, oxidative stress, and fibrosis of the nephron can be avoided by preventing or correcting gut dysbiosis to help prevent CKD progression therefore appears appealing. The administration of oral FMT in patients with CKD has potential benefits, and further research is required in this area.

## Figures and Tables

**Table 1:** Comparison of baseline characteristics between CKD patients from the FMT group and the placebo group.

Characteristic	FMT (n = 15) n (% or SD)	Placebo (n = 13) n (% or SD)	*p* Value
Demographic data			
Age in years (range)	57 (44–71)	56 (32–76)	ND
Male gender	8 (61.5)	6 (40.0)	ND
Etiology			
Type 2 diabetes	14 (93.3)	11 (84.6)	ND
Hypertension	1 (6.6)	2 (15.3)	ND
CKD stages (GFR, mL/min per 1.73 m^2^)			
G1 (>90)	0 (0.0)	1 (7.14)	0.48
G2 (60–89)	2 (12.5)	1 (7.14)	0.99
G3a (45–59)	2 (12.5)	3 (21.4)	0.63
G3b (30–44)	3 (25.0)	2 (14.2)	0.99
G4 (15–29)	6 (37.5)	6 (50.0)	0.99
G5 (<15)	2 (12.5)	0 (0.0)	0.48
Albuminuria stages (ACR, mg)			
A2 (30–300)	6 (37.5)	6 (50.0)	0.99
A3 (>300)	9 (62.5)	7 (50.0)	0.99
Laboratory results			
Hemoglobin, g/dL	11.02 (±1.80)	11.46 (±1.96)	0.54
Leukocytes, K/μL	8.72 (±2.21)	6.81 (±1.67)	**0.01**
Platelets, K/μL	227.00 (±93.50)	205.53 (±49.44)	0.44
Glucose, mg/dL	130.93 (±51.07)	123.76 (±73.30)	0.76
Urine protein, g/24 h	2.05 (±2.68)	1.67 (±2.04)	0.68
Creatinine clearance, mL/min	32.41 (±15.84)	42.15 (±25.71)	0.23
Blood urea nitrogen, mg/dL	38.93 (±15.90)	32.76 (±14.10)	0.29
Creatinine, mg/dL	2.30 (±0.62)	2.26 (±0.75)	0.86
Uric acid, mg/dL	7.79 (±1.79)	6.27 (±1.56)	**0.02**
C-reactive protein, mg/dL	0.79 (±1.18)	0.54 (±0.08)	0.44
Potassium, mmol/L	5.14 (±0.77)	4.97 (±0.64)	0.53
Phosphorus, mg/dL	4.20 (±0.39)	4.14 (±0.88)	0.82
HCO_3_, mEq/L	22.98 (±3.25)	26.32 (±2.77)	**0.008**
Adverse events			
Abdominal distention	5 (33.3)	0 (0.0)	ND
Diarrhea	3 (20.0)	4 (30.8)	ND
Constipation	2 (13.3)	3 (23.1)	ND
Increased frequency of bowel movements	2 (13.3)	0 (0.0)	ND
Flatulence	1 (6.7)	0 (0.0)	ND
Fever	0 (0.0)	1 (7.7)	ND

ACR: albumin-creatinine ratio; CKD: chronic kidney disease; FMT: fecal microbiota transplant; GFR: glomerular filtration rate; ND: not determined; SD: standard deviation. Statistically significant differences are marked in bold letters.

**Table 2:** Comparison of mean relative abundance of *Roseburia* spp. between fecal microbiota transplant (FMT) treatment and placebo groups. Tukey (HSD) post hoc analysis for *Roseburia* spp. is presented with a mean relative abundance > 0.01% and a significant difference (*p* < 0.05) between at least two groups, which are marked in bold letters.

Group	Day	Mean Relative Abundance	Fecal Microbiota Transplant (FMT)					
			Day 0		Day 30		Day 90	
			Standardized Difference	*p* Value	Standardized Difference	*p* Value	Standardized Difference	*p* Value
Placebo	0	2.716	9.850	**<0.0001**	15.583	**<0.0001**	0.640	0.987
	30	2.546	11.044	**<0.0001**	16.777	**<0.0001**	1.834	0.458
	90	7.251	22.097	**<0.0001**	16.363	**<0.0001**	31.307	**<0.0001**
FMT	0	4.114	ND	ND	0.640	0.987	9.210	**<0.0001**
	30	4.928	ND	ND	ND	ND	14.943	**<0.0001**
	90	2.807	ND	ND	ND	ND	ND	ND

FMT: fecal microbiota transplant; ND: not determined.

## Data Availability

The original contributions presented in the study are included in the article/Supplementary Material; further inquiries can be directed to the corresponding author.

## Supplementary Materials

The following supporting information can be downloaded at: https://www.mdpi.com/article/10.3390/nu16081109/s1, Figure S1: Alpha diversity analysis of gut microbiome after FMT treatment; Table S1: Evolution of biochemical parameters of CKD patients from the FMT group and the placebo group.
