# Supplementary material for: Changes in the Progression of Chronic Kidney Disease in Patients Undergoing Fecal Microbiota Transplantation
Source: Nutrients. 2024 Apr 10;16(8):1109. doi: 10.3390/nu16081109 (PMC11055146; doi:10.3390/nu16081109)

**Supplementary Figure S1. Alpha diversity analysis of gut microbiome of after FMT treatment** Stool samples from patients after fecal microbiota transplantation (FMT) or placebo treatments were assessed by metagenomic analysis. Alpha diversity of samples was assessed using comparisons of Amplicon Sequence Variants (ASVs) and Shannon diversity indices. **a.** Observed features (ASVs) boxplot. According to the number of ASVs, the microbial diversity between FMT and placebo groups was not significantly different ($p > 0.05$). **b.** Shannon diversity boxplot. The analysis of the Shannon diversity indices showed that the microbial diversity between FMT and placebo groups was not significantly different ($p > 0.05$).

a

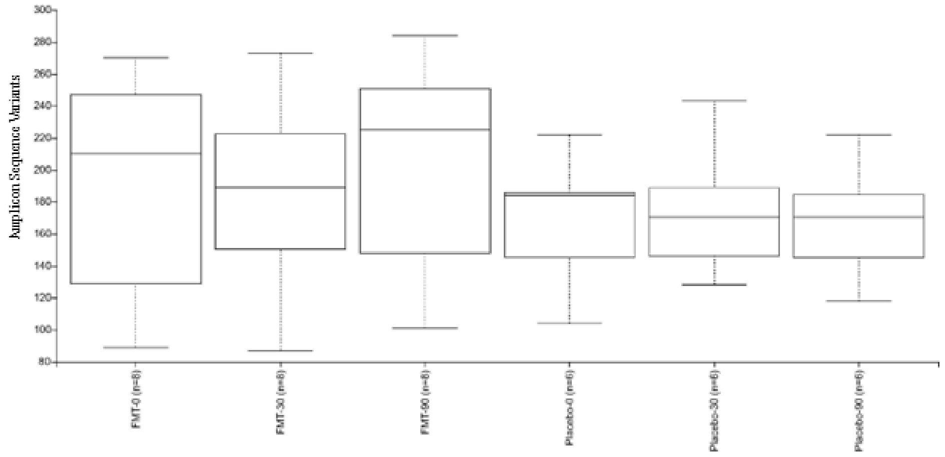


b

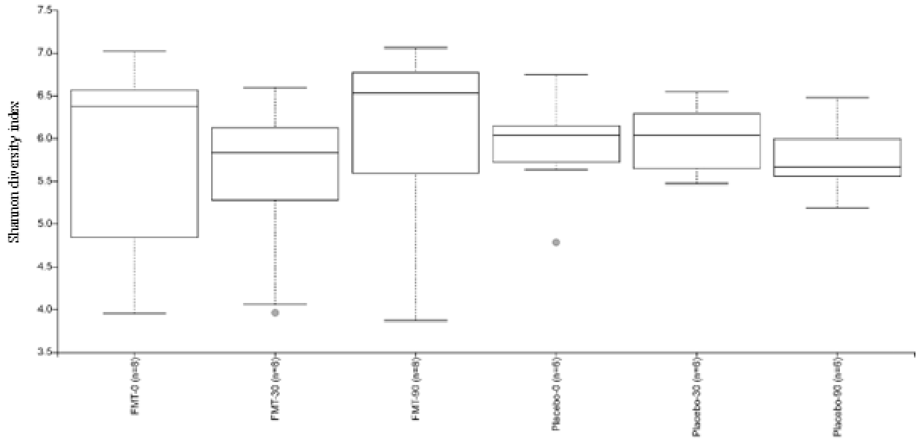

Supplement: Supplementary file 1 [file nutrients-16-01109-s001.zip › Figure S1.pdf]
