# Supplementary material for: Changes in the Progression of Chronic Kidney Disease in Patients Undergoing Fecal Microbiota Transplantation
Source: Nutrients. 2024 Apr 10;16(8):1109. doi: 10.3390/nu16081109 (PMC11055146; doi:10.3390/nu16081109)
Supplement: Supplementary file 1 [file nutrients-16-01109-s001.zip › Table S1.pdf]

**Supplementary Table S1.** Evolution of biochemical parameters of CKD patients from the FMT group and the placebo group

| Days after treatment | **FMT** (*n*= 15) Mean (SD) | **Placebo** (*n*= 13) Mean (SD) | ***p* value** |
|---|---|---|---|
| | **Hemoglobin, g/dL** | | |
| Day 10 | 11.02 (± 1.98) | 11.33 (± 1.82) | 0.67 |
| Day 30 | 10.98 (± 1.95) | 11.42 (± 1.91) | 0.55 |
| Day 60 | 10.91 (± 1.73) | 11.50 (± 1.95) | 0.41 |
| Day 90 | 11.25 (± 1.30) | 11.56 (± 1.86) | 0.60 |
| Day 120 | 11.32 (± 1.32) | 11.92 (± 1.65) | 0.31 |
| Day 180 | 11.41 (± 1.83) | 11.58 (± 1.64) | 0.80 |
| *p* value | 0.91 | 0.40 | |
| | **Leukocytes, K/uL** | | |
| Day 10 | 8.22 (± 1.41) | 6.89 (± 1.46) | **0.02** |
| Day 30 | 8.46 (± 1.93) | 6.72 (± 1.24) | **0.01** |
| Day 60 | 8.67 (± 1.83) | 7.17 (± 2.28) | 0.06 |
| Day 90 | 8.09 (± 1.55) | 7.19 (± 1.56) | 0.15 |
| Day 120 | 8.26 (± 1.92) | 6.74 (± 1.49) | **0.03** |
| Day 180 | 8.65 (± 2.23) | 7.23 (± 1.30) | 0.05 |
| *p* value | 0.72 | 0.63 | |
| | **Platelets, K/uL** | | |
| Day 10 | 222.64 (± 82.13) | 201.62 (± 43.02) | 0.41 |
| Day 30 | 235.80 (± 81.78) | 210.77 (± 51.19) | 0.35 |
| Day 60 | 223.07 (± 94.85) | 201.77 (± 39.73) | 0.45 |
| Day 90 | 224.46 (± 62.31) | 205.31 (± 51.70) | 0.40 |
| Day 120 | 218.69 (± 64.25) | 204.39 (± 40.48) | 0.50 |
| Day 180 | 218.92 (± 68.64) | 215.00 (± 46.87) | 0.86 |
| *p* value | 0.30 | 0.29 | |
| | **Glucose, mg/dL** | | |
| Day 10 | 111.20 (± 31.04) | 96.23 (± 18.59) | 0.13 |
| Day 30 | 111.27 (± 34.18) | 94.54 (± 14.26) | 0.10 |
| Day 60 | 110.13 (± 46.79) | 108.08 (± 28.43) | 0.89 |
| Day 90 | 123.39 (± 30.71) | 99.54 (± 26.61) | **0.04** |
| Day 120 | 123.23 (± 62.08) | 119.77 (± 53.43) | 0.88 |
| Day 180 | 127.23 (± 44.71) | 94.85 (± 13.63) | **0.02** |
| *p* value | 0.61 | 0.29 | |
| | **Urine protein, g/24 h** | | |
| Day 10 | 1.96 (± 2.44) | 1.74 (± 2.17) | 0.80 |
| Day 30 | 2.20 (± 2.40) | 1.33 (± 1.74) | 0.28 |
| Day 60 | 2.35 (± 2.55) | 1.71 (± 1.95) | 0.46 |
| Day 90 | 2.14 (± 2.02) | 1.99 (± 2.17) | 0.85 |
| Day 120 | 2.54 (± 2.25) | 1.42 (± 1.32) | 0.13 |

| | | | |
|---|---|---|---|
| Day 180 | 3.01 (± 2.73) | 2.34 (± 2.14) | 0.49 |
| *p* value | 0.44 | 0.27 | |
| **Creatinine clearance, mL/min** | | | |
| Day 10 | 35.73 (± 17.17) | 40.92 (± 24.00) | 0.51 |
| Day 30 | 35.83 (± 20.09) | 40.00 (± 19.36) | 0.58 |
| Day 60 | 37.40 (± 20.45) | 41.15 (± 21.93) | 0.64 |
| Day 90 | 40.41 (± 18.90) | 42.15 (± 19.04) | 0.81 |
| Day 120 | 41.23 (± 20.07) | 46.69 (± 29.01) | 0.58 |
| Day 180 | 41.23 (± 20.24) | 42.08 (± 25.88) | 0.92 |
| *p* value | 0.25 | 0.44 | |
| **Blood urea nitrogen, mg/dL** | | | |
| Day 10 | 40.93 (± 15.67) | 30.31 (± 9.87) | **0.04** |
| Day 30 | 37.73 (± 13.73) | 30.62 (± 10.62) | 0.13 |
| Day 60 | 40.40 (± 18.93) | 34.00 (± 14.58) | 0.33 |
| Day 90 | 36.69 (± 15.77) | 34.62 (± 15.22) | 0.76 |
| Day 120 | 38.85 (± 18.42) | 32.31 (± 14.12) | 0.32 |
| Day 180 | 35.00 (± 14.20) | 32.15 (± 11.52) | 0.58 |
| *p* value | 0.51 | 0.52 | |
| **Creatinine, mg/dL** | | | |
| Day 10 | 2.35 (± 0.87) | 2.13 (± 0.59) | 0.44 |
| Day 30 | 2.20 (± 0.80) | 2.20 (± 0.61) | 0.99 |
| Day 60 | 2.41 (± 0.99) | 2.32 (± 0.74) | 0.79 |
| Day 90 | 2.12 (± 0.89) | 2.22 (± 0.77) | 0.77 |
| Day 120 | 2.31 (± 1.21) | 2.26 (± 0.85) | 0.91 |
| Day 180 | 2.34 (± 1.09) | 2.35 (± 0.91) | 0.98 |
| *p* value | 0.47 | 0.31 | |
| **Uric acid, mg/dL** | | | |
| Day 10 | 7.09 (± 1.46) | 6.24 (± 1.63) | 0.15 |
| Day 30 | 6.99 (± 1.46) | 6.59 (± 1.93) | 0.53 |
| Day 60 | 7.00 (± 1.65) | 6.95 (± 1.30) | 0.93 |
| Day 90 | 6.92 (± 1.38) | 6.57 (± 1.46) | 0.54 |
| Day 120 | 6.77 (± 1.36) | 7.13 (± 1.21) | 0.48 |
| Day 180 | 6.21 (± 1.04) | 7.28 (± 1.53) | **0.04** |
| *p* value | **0.02** | 0.15 | |
| **C-reactive protein, mg/dL** | | | |
| Day 10 | 0.80 (± 0.68) | 0.51 (± 0.03) | 0.15 |
| Day 30 | 0.98 (± 0.91) | 0.63 (± 0.30) | 0.20 |
| Day 60 | 0.99 (± 0.94) | 1.05 (± 1.94) | 0.91 |
| Day 90 | 0.71 (± 0.26) | 0.62 (± 0.16) | 0.33 |
| Day 120 | 0.88 (± 0.49) | 0.57 (± 0.14) | **0.04** |
| Day 180 | 0.72 (± 0.28) | 0.56 (± 0.14) | 0.08 |
| *p* value | 0.17 | 0.38 | |
| **Potassium, mmol/L** | | | |

| | | | |
|---|---|---|---|
| Day 10 | 4.81 (± 0.64) | 4.72 (± 0.69) | 0.72 |
| Day 30 | 4.99 (± 0.75) | 4.89 (± 0.68) | 0.69 |
| Day 60 | 5.04 (± 0.47) | 5.15 (± 0.93) | 0.69 |
| Day 90 | 5.20 (± 0.60) | 4.86 (± 0.87) | 0.26 |
| Day 120 | 5.02 (± 0.59) | 4.79 (± 0.61) | 0.33 |
| Day 180 | 5.25 (± 0.65) | 5.08 (± 0.87) | 0.56 |
| *p* value | 0.41 | 0.41 | |
| | **Phosphorus, mg/dL** | | |
| Day 10 | 4.48 (± 0.57) | 4.02 (± 0.73) | 0.07 |
| Day 30 | 4.25 (± 0.59) | 4.40 (± 0.90) | 0.61 |
| Day 60 | 4.48 (± 0.39) | 4.39 (± 0.87) | 0.72 |
| Day 90 | 4.35 (± 0.72) | 4.25 (± 0.75) | 0.73 |
| Day 120 | 4.26 (± 0.62) | 4.23 (± 0.81) | 0.91 |
| Day 180 | 4.22 (± 0.38) | 4.39 (± 0.86) | 0.54 |
| *p* value | 0.40 | 0.05 | |
| | **$HCO_3$, mEq/L** | | |
| Day 10 | 22.44 (± 4.37) | 24.52 (± 3.00) | 0.16 |
| Day 30 | 21.97 (± 4.25) | 22.53 (± 3.88) | 0.71 |
| Day 60 | 20.99 (± 2.55) | 24.46 (± 2.69) | **0.002** |
| Day 90 | 20.84 (± 2.84) | 22.20 (± 6.80) | 0.49 |
| Day 120 | 21.84 (± 4.28) | 24.40 (± 3.04) | 0.09 |
| Day 180 | 20.85 (± 3.59) | 24.54 (± 3.03) | **0.01** |
| *p* value | 0.36 | 0.14 | |

SD: standard deviation. Statistically significant differences are remarked in bold letters.
